# Warburg Effect Presenting as Refractory Type B Lactic Acidosis: A Pediatric Case Report

**DOI:** 10.7759/cureus.117952

**Published:** 2026-10-15

**Authors:** Amjad M Ghandurah, Bashaer Baharoon, Rahaf Almuhanna, Enass H Raffa, Amal A Alharbi, Dalal Althubaiti, Anas F Galleon

**Affiliations:** 1 Pediatric Critical Care Medicine, King Faisal Specialist Hospital and Research Centre, Jeddah, SAU; 2 Oncology, King Faisal Specialist Hospital and Research Centre, Jeddah, SAU; 3 Emergency Medicine, King Faisal Specialist Hospital and Research Centre, Jeddah, SAU

**Keywords:** acute lymphoblastic leukemia (all), lactic acid, pediatric intensive care unit (picu), severe acidosis, warburg effect

## Abstract

Type B lactic acidosis is a rare but life-threatening metabolic complication of hematologic malignancies, attributed to the Warburg effect, in which tumor cells preferentially generate energy through aerobic glycolysis despite adequate oxygen availability. Although increasingly recognized in adults, pediatric cases remain uncommon and are associated with high mortality.

A 12-year-old boy with early isolated marrow relapse of high-risk precursor B acute lymphoblastic leukemia (B-ALL) developed rapidly progressive lactic acidosis and recurrent hypoglycemia during re-induction chemotherapy. Despite extensive investigations, no alternative etiology was identified, including hypoxemia, endocrinopathy, inborn errors of metabolism, or significant hepatic or renal dysfunction at the onset of lactic acidosis. Repeated microbiological investigations remained negative throughout the clinical course. Repeat bone marrow aspiration and biopsy demonstrated refractory leukemia with 70% blasts, supporting a diagnosis of malignancy-associated type B lactic acidosis consistent with the Warburg effect. Despite aggressive supportive care, corticosteroid cytoreduction, and subsequent anti-CD22-targeted therapy with inotuzumab, the metabolic derangement persisted in parallel with progressive leukemia, resulting in multiorgan dysfunction and death. This case highlights that persistent lactic acidosis with refractory hypoglycemia arising during re-induction therapy may be an early indicator of refractory disease rather than treatment-related toxicity or occult infection. Malignancy-associated type B lactic acidosis should be considered after more common etiologies have been excluded. Early recognition may facilitate timely initiation of definitive anti-leukemic therapy, which remains the cornerstone of management despite the overall poor prognosis.

## Introduction

Type B lactic acidosis is an uncommon but well-recognized metabolic complication of hematologic malignancies that occurs independently of tissue perfusion or hypoxia and represents a distinct clinicopathologic entity from the more frequently encountered type A lactic acidosis. It has been described as being associated with acute leukemias and aggressive lymphomas, where prompt recognition is essential as definitive management relies primarily on effective cytoreduction rather than supportive measures alone [1,2].

The metabolic basis of malignancy-associated type B lactic acidosis is most commonly attributed to the Warburg effect, a hallmark of cancer metabolism in which proliferating malignant cells preferentially utilize aerobic glycolysis to sustain cellular growth despite sufficient oxygen availability [3]. This metabolic reprogramming, first described by Warburg in the early 20th century, promotes lactate production and accelerated glucose utilization, explaining the characteristic association of profound hyperlactatemia with refractory hypoglycemia observed in a subset of patients [4,5].

Pediatric experience with malignancy-associated type B lactic acidosis remains limited. The largest published systematic review by Permtawee et al. identified 20 reported cases, predominantly in acute lymphoblastic leukemia and non-Hodgkin lymphoma, with relapsed disease consistently associated with the poorest outcomes [6]. Despite growing recognition of this syndrome, the heterogeneity of clinical presentation may pose challenges for timely diagnosis and therapeutic decision-making.

We report a child with early isolated marrow relapse of precursor B-acute lymphoblastic leukemia (B-ALL) who developed severe type B lactic acidosis with recurrent hypoglycemia during re-induction chemotherapy. This case is discussed in the context of published pediatric literature, with particular emphasis on the occurrence of this metabolic syndrome during treatment for relapsed leukemia and its relationship to refractory disease.

Unlike most previously published pediatric cases, which primarily involve newly diagnosed or myeloid leukemias, this report specifically describes the Warburg-associated metabolic syndrome arising during re-induction therapy for early isolated marrow relapse of B-ALL, a particularly rare and under-reported clinical scenario.

## Case presentation

A 12-year-old male with high-risk hypodiploid B-ALL, CNS 1, was initially diagnosed in November 2024 and treated according to the Children’s Oncology Group (COG) AALL1732 high-risk protocol. He achieved first complete remission with end-of-induction minimal residual disease negativity (<0.01%). His treatment course was complicated by transient steroid- and asparaginase-induced hyperglycemia requiring temporary insulin therapy. After discharge, the patient was clinically well.

While awaiting hematologic recovery following delayed intensification, the patient presented with persistent high-grade fever (maximum temperature: 39.5°C; blood pressure {BP}: 125/73 mmHg; mean arterial pressure {MAP}: 91 mmHg; heart rate {HR}: 148 beats/min; respiratory rate {RR}: 24 breaths/min) without localizing symptoms. Initial physical examination was unremarkable. Laboratory investigations revealed mild thrombocytopenia, 3% circulating peripheral blasts, ferritin 10,450 µg/L, C-reactive protein 176 mg/L, and procalcitonin 9.01 ng/mL (Table 1). A comprehensive infectious evaluation was performed, including blood, cerebrospinal fluid, and urine cultures, respiratory viral multiplex PCR, investigations for invasive fungal disease, chest computed tomography (CT), and abdominal ultrasonography. All investigations were negative, and no infectious source was identified, but nine days after admission the patient was noted to have elevated lactic acid (11.7 mmol/L). Despite empiric broad-spectrum antibiotics and antifungal therapy, his clinical condition failed to improve. Bone marrow aspiration subsequently confirmed early isolated medullary relapse of B-ALL, and re-induction chemotherapy was initiated according to the COG AALL1331 protocol. During re-induction, counts began to recover; however, reappearance of peripheral blasts raised concern for refractory disease.

**Table 1:** Evolution of laboratory parameters during the clinical course. LDH: lactate dehydrogenase; ALT: alanine aminotransferase; AST: aspartate aminotransferase

Parameters (unit)	Published reference value	Initial presentation	Marrow relapse (day 9)	Metabolic syndrome: re-induction day 22	Peak metabolic deterioration (day 34)
WBC (×10⁹/L)	3.8-10.4 [7]	8.26	24.46	1.18	0.49
Hemoglobin (g/L)	124-157 [7]	100	89	77	74
Platelets (×10⁹/L)	177-381 [7]	103	27	17	3
Peripheral blasts (%)	0 (absent) [8]	3	2	66	-
LDH (U/L)	120-293 [7]	642	1,399	855	>1,800
Glucose (mmol/L)	3.6-7.7 [9]	6.4	5.2	5.5	4.3
Lactate (mmol/L)	≤2.2 (plasma) [7]	-	11.7	18.3	Above assay detection limit
pH	7.35-7.45 (arterial); 7.33-7.43 (venous); 7.30-7.40 (capillary) [9]	-	7.31	7.31	7.14
Bicarbonate (mmol/L)	21-29 (serum) [7]; 22-26 (arterial); 23-27 (venous) [10]	17.1	14.4	13.4	9.6
ALT (U/L)	7-55 [7]	25	16	31	590.0
AST (U/L)	8-60 [7]	57	81	55	118
Creatinine (µmol/L)	26.5-79.6 [9]	38	31	37	57
Total bilirubin (µmol/L)	0-17.1 [7]	7.5	14.6	17.8	39.3
Ferritin (µg/L)	15-201 [7]	10,451	13,620	-	-
CRP (mg/L)	<5.0 [7]	176.0	-	343.00	676.0
Procalcitonin (ng/mL)	≤0.1 [11]	9.01	-	49.60	40.50

During re-induction, transient corticosteroid-induced hyperglycemia required temporary basal-bolus insulin therapy. On re-induction day 22, the patient developed recurrent hypoglycemia, associated with progressive hyperlactatemia and high anion gap metabolic acidosis. Insulin was discontinued, and intravenous dextrose 10% boluses together with sodium bicarbonate replacement were initiated. At the onset of these metabolic abnormalities, he remained asymptomatic apart from persistent fever, with preserved hemodynamic status, adequate oxygenation and perfusion, a normal physical examination, and normal renal and hepatic function.

The following day, the patient developed lethargy, hypotension (temperature {T}: 39.4°C; respiratory rate {RR}: 42 breaths/min; heart rate {HR}: 133 beats/min; blood pressure {BP}: 90/49 mmHg), and persistent metabolic acidosis with progressive hyperlactatemia, prompting transfer to the pediatric intensive care unit (PICU). Broad-spectrum antimicrobial therapy was escalated to meropenem, vancomycin, and caspofungin with continuation of *Pneumocystis jirovecii* prophylaxis. Stress-dose hydrocortisone and continuous dextrose 10% infusion were initiated. Scheduled day 22 chemotherapy (vincristine and the third dose of Erwinia asparaginase) was withheld because of his clinical deterioration.

Repeated microbiological investigations remained negative. Serum biochemistry, including renal and hepatic function, remained within normal limits during the initial phase of metabolic deterioration. A critical sample obtained during hypoglycemia demonstrated an appropriate counter-regulatory cortisol response (morning cortisol: 792 nmol/L) with suppressed insulin secretion (insulin: 2.78 pmol/L; C-peptide: 0.854 nmol/L), excluding adrenal insufficiency and endogenous hyperinsulinism. Computed tomography of the brain and transthoracic echocardiography were unremarkable. In the absence of tissue hypoperfusion, significant hepatic or renal dysfunction, endocrinopathy, or an identifiable infectious source, the constellation of refractory hypoglycemia, persistent metabolic acidosis, and progressive lactic acidosis was considered most consistent with malignancy-associated type B lactic acidosis (Warburg effect).

Within 48 h of developing refractory hyperlactatemia and hypoglycemia, methylprednisolone (40 mg/m^2^/day intravenously in two divided doses) together with intravenous thiamine (100 mg daily) was initiated for cytoreduction and metabolic support. Repeat bone marrow aspiration and biopsy demonstrated persistent disease with 70% leukemic blasts expressing CD19 and CD22, confirming refractory B-ALL. Salvage therapy with the anti-CD22 monoclonal antibody inotuzumab ozogamicin was initiated five days after the onset of metabolic deterioration (re-induction day 27).

Despite initiation of salvage therapy, the patient’s condition continued to deteriorate with progressive respiratory distress, generalized edema, pulmonary edema, pericardial effusion, moderate ascites, and hepatosplenomegaly. Veno-occlusive disease (VOD) was considered due to the refractory thrombocytopenia, weight gain, hepatomegaly, and ascites; however, Doppler ultrasonography demonstrated normal hepatic vasculature.

On day 32, he developed a decline in level of consciousness (Glasgow Coma Scale 6/15) and hemodynamic instability requiring endotracheal intubation, mechanical ventilation, and inotropic support. Comprehensive neurological evaluation, including CT, magnetic resonance imaging/angiography/venography of the brain and spine, and electroencephalogram (EEG), showed no acute intracranial abnormality to account for his neurological deterioration.

The patient’s clinical course was further complicated by progressive multiorgan dysfunction. Despite maximal supportive care, including vasopressor therapy and continuous renal replacement therapy (continuous renal replacement therapy {CRRT}, specifically continuous venovenous hemodiafiltration {CVVHDF}), hyperlactatemia, metabolic acidosis, and hypoglycemia remained refractory (Table 1). Following multidisciplinary discussions with the family, goals of care were redirected to comfort-focused management, and the patient died on day 38 (Figures 1, 2).

**Figure 1:**
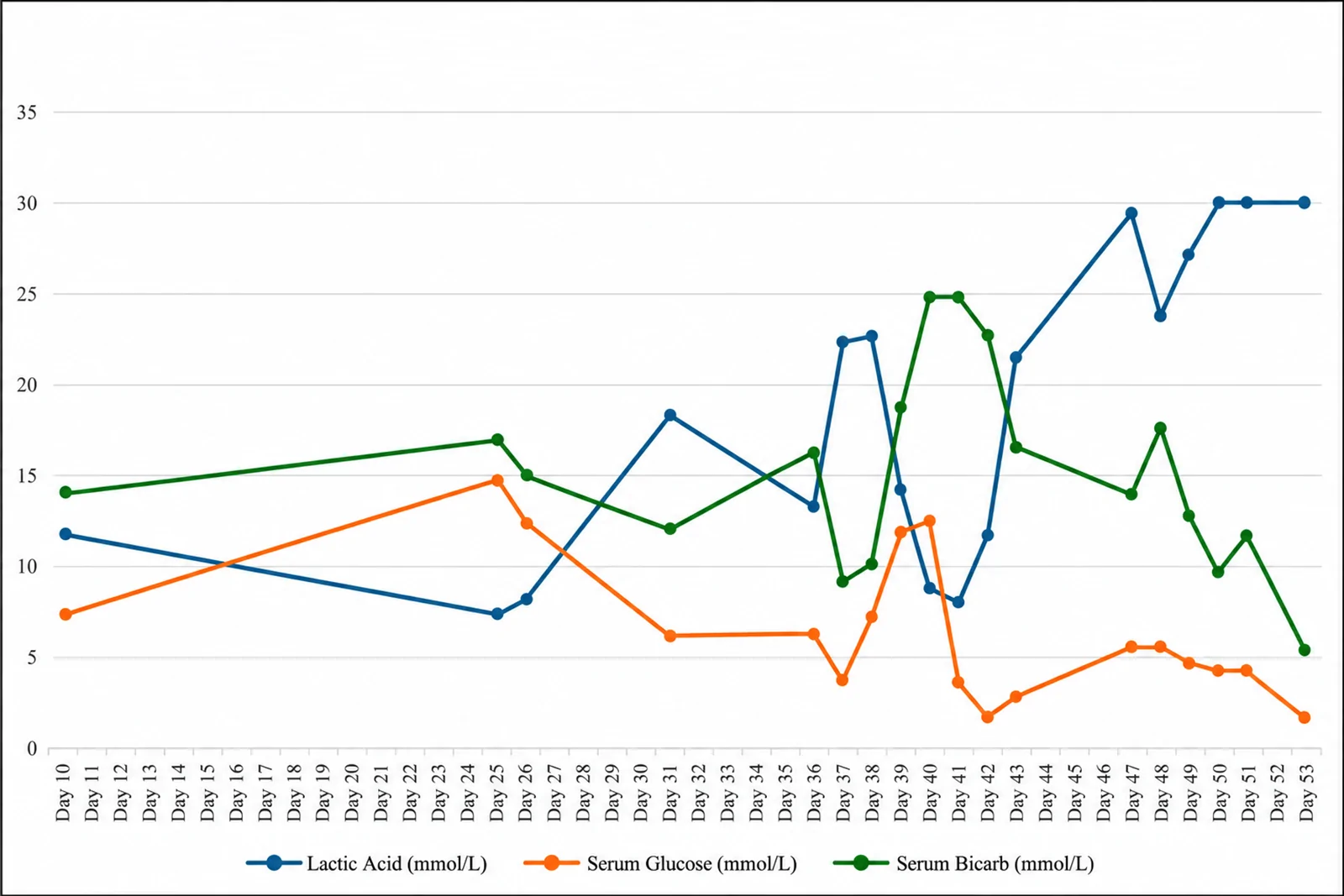
Trends in lactate, glucose, and bicarbonate during the clinical course. Serial lactate, glucose, and bicarbonate concentrations during the clinical course of relapsed B-cell acute lymphoblastic leukemia complicated by suspected malignancy-associated type B lactic acidosis. All concentrations are expressed in mmol/L; the horizontal axis shows the sampling dates. The plotted values should be interpreted alongside the clinical chronology and the selected laboratory measurements in Table 1.

**Figure 2:**
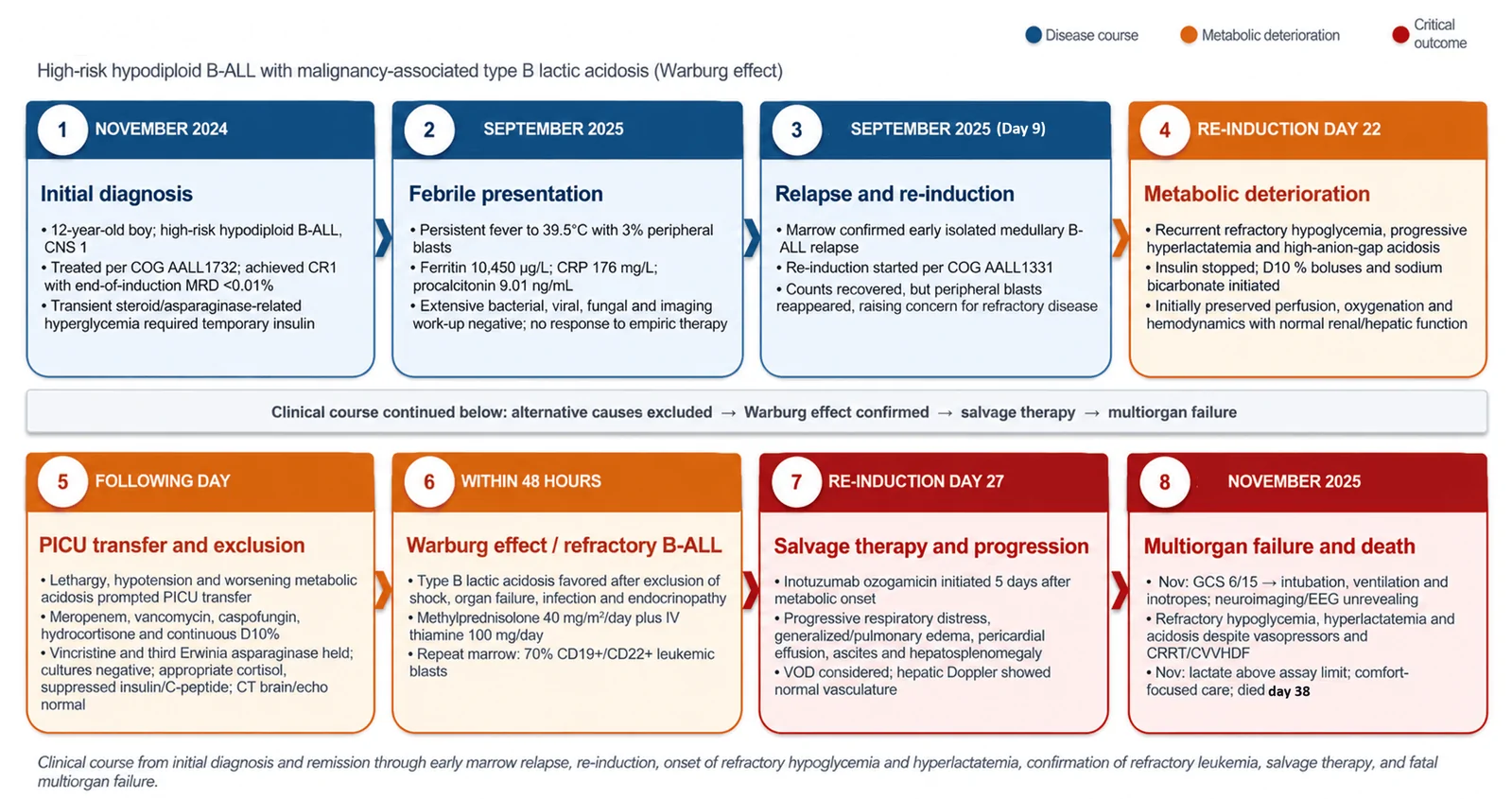
Clinical timeline of relapsed B-cell acute lymphoblastic leukemia complicated by suspected malignancy-associated type B lactic acidosis. Timeline from the initial diagnosis in November 2024 through marrow relapse in September 2025, re-induction chemotherapy, refractory hypoglycemia and hyperlactatemia, confirmation of refractory leukemia with 70% marrow blasts, salvage therapy with inotuzumab ozogamicin, and subsequent multiorgan dysfunction and death in November 2025. B-ALL: B-cell acute lymphoblastic leukemia; COG: Children’s Oncology Group; CR1: first complete remission; MRD: minimal residual disease; PICU: pediatric intensive care unit; D10%: 10% dextrose; CD: cluster of differentiation; VOD: veno-occlusive disease; GCS: Glasgow Coma Scale; CRRT: continuous renal replacement therapy; CVVHDF: continuous venovenous hemodiafiltration

## Discussion

In children with acute leukemia, the diagnosis of malignancy-associated type B lactic acidosis requires systematic exclusion of more common causes of hyperlactatemia before the metabolic derangement can be attributed to the underlying malignancy. At the onset of metabolic deterioration, our patient had preserved tissue perfusion, adequate oxygenation, and no evidence of hemodynamic instability or shock. Hepatic and renal function were also preserved, making type A lactic acidosis caused by tissue hypoxia or systemic hypoperfusion, as well as impaired lactate clearance, unlikely. Subsequent bone marrow examination demonstrating 70% leukemic blasts despite three weeks of re-induction chemotherapy further supported refractory leukemia as the underlying driver of the metabolic derangement [12].

Published pediatric experience with malignancy-associated type B lactic acidosis remains limited and is summarized in Table 2. The systematic review by Permtawee et al. represents the most comprehensive synthesis of the available evidence and demonstrated that relapsed disease was consistently associated with poor outcomes [6]. Importantly, normalization of lactate was observed predominantly in patients who achieved effective cytoreduction, reinforcing that treatment of the underlying malignancy, rather than correction of the metabolic abnormalities alone, is essential for reversal of the metabolic derangement. These observations are further supported by subsequent pediatric case reports, which consistently describe hyperlactatemia with refractory hypoglycemia as markers of aggressive disease biology and poor prognosis [13-15].

**Table 2:** Published cases of malignancy-associated type B lactic acidosis in children and adolescents. NR: not reported; M: male; F: female; ALL: acute lymphoblastic leukemia; AKI: acute kidney injury; BL: Burkitt lymphoma; B-NHL: B-cell non-Hodgkin lymphoma; T-NHL: T-cell non-Hodgkin lymphoma; PICU: pediatric intensive care unit; CRRT: continuous renal replacement therapy; CVVH: continuous venovenous hemofiltration; HSCT: hematopoietic stem cell transplantation; BFM: Berlin-Frankfurt-Münster; COALL: German Cooperative Study Group for Childhood Acute Lymphoblastic Leukemia; EICNHL: European Intergroup for Childhood Non-Hodgkin Lymphoma

Studies	Age (years)/sex	Diagnosis	Disease status	Presenting features	Peak lactate (mmol/L)	Reported glucose (mg/dL)	Alternative causes excluded	Supportive therapy	Disease-directed therapy	Outcome
Permtawee et al. (2023) [6]	10/M	Burkitt leukemia	Newly diagnosed	Poor intake with hypoglycemia and lactic acidosis	3.7	49	Yes	Bicarbonate, hydration, allopurinol, transfusion	Rituximab-containing multiagent chemotherapy for Burkitt leukemia	Achieved complete remission; lactic acidosis resolved after initiation of chemotherapy
Narayani et al. (2019) [13]	2/M	ALL	Newly diagnosed	Fever; cachectic and pale with pancytopenia, and metabolic acidosis	13.5	96	Yes	Transfusions, hydration, bicarbonate	Induction chemotherapy	Lactate progressively decreased after chemotherapy initiation
Vagha et al. (2024) [14]	14/F	B-cell precursor ALL	Newly diagnosed	Fever, leg pain, abdominal pain, hepatosplenomegaly, anemia, thrombocytopenia, tachypnea	12.0	NR	Yes	Packed RBC transfusion, platelet transfusions, intravenous sodium bicarbonate infusion followed by oral sodium bicarbonate	BFM-95 induction chemotherapy	Lactate and metabolic acidosis improved approximately 30 h after chemotherapy initiation; patient showed marked clinical improvement and survived induction
Yıldırım et al. (2026) [15]	13/M	T-cell lymphoblastic lymphoma	Newly diagnosed	Type B lactic acidosis, AKI	10.1	61	NR	NR	Chemotherapy	Improved after initiation of therapy. However, he died later during treatment from septic shock
Kharayat et al. (2024) [16]	17/M	T-cell ALL	Newly diagnosed	Vomiting, poor intake, body aches, headache, abdominal symptoms; severe anion gap metabolic acidosis with persistent hyperlactatemia	18.0	60	Yes	IV fluids, empiric antibiotics initially, acetate-containing fluids, PICU supportive care	Chemotherapy	Rapid improvement and resolution of lactic acidosis after chemotherapy initiation
Müngen et al. (2024) [17]	16/F	Burkitt lymphoma (primary breast BL)	Newly diagnosed, stage IV	Bilateral breast swelling, respiratory distress, severe lactic acidosis	22	NR	NR	Dialysis support	EICNHL Mature B-NHL protocol, Group C3 chemotherapy	Very good partial response; lactate normalized after chemotherapy. Died 3 months later due to fungal septicemia
Sillos et al. (2001) [18]	11/F	T-cell ALL	Third relapse	Altered mental status, circulating blasts, severe metabolic acidosis	15.5	47	Yes	Bicarbonate, CVVH	Salvage chemotherapy	Transient lactate improvement; died 23 days later
Sillos et al. (2001) [18]	17/M	T-cell ALL	Relapsed/refractory disease	Somnolence, tachypnea, severe metabolic acidosis	23.0	132	Yes	Bicarbonate, supportive care	No effective salvage therapy	Rapid deterioration; died 10 h after recognition
Sillos et al. (2001) [18]	18/F	T-NHL	Newly diagnosed, stage IV	Weight loss, fever, lymphadenopathy, pleural effusion, metabolic acidosis	16.3	44	Yes	Glucose, bicarbonate	Steroids + cyclophosphamide + vincristine	Lactate improved after chemotherapy; later relapse and death
Hui et al. (2021) [19]	17/M	B-cell precursor ALL	Relapsed	Poor appetite, weight loss, bicytopenia	18.0	NR	NR	Bicarbonate, and 3 sessions of CRRT	NR	Lactate level decreased following CRRT; outcome of leukemia treatment not reported in detail
Khera et al. (2020) [20]	11/F	T-cell ALL	Newly diagnosed	Fever, vomiting, abdominal pain, and respiratory distress	20.0	104.4	Yes	Transfusions, bicarbonate	Chemotherapy as per BFM	Lactate progressively decreased after chemotherapy initiation
Schuh et al. (2018) [21]	12/M	Pre-B-cell ALL	Relapsed/refractory	Distress, tachycardia, fever, diffuse abdominal tenderness, bicytopenia; severe metabolic acidosis	14.8	80	Yes	Aggressive IV fluid resuscitation; empiric IV antibiotics	Chemotherapy	Lactic acidosis worsened; died from progressive disease
Gökçe et al. (2012) [22]	13/M	ALL	Newly diagnosed	Weight loss, fatigue, bone pain, severe metabolic acidosis; hepatomegaly and nephromegaly	61.2	97	Yes	Transfusions, hydration, bicarbonate	Induction chemotherapy	Lactic acidosis resolved after chemotherapy; achieved remission
Terpe et al. (2012) [23]	11/M	B-cell precursor ALL	Newly diagnosed	Nausea, vertigo, dyspnea, and chest pain with hepatosplenomegaly and lymphadenopathy	40	48.6	Yes	Intensive supportive care	ALL-directed chemotherapy (COALL-07-03)	Died 6 days after initial admission due to multiorgan failure
Kulkarni et al. (2010) [24]	12/M	Burkitt lymphoma (BL)	Newly diagnosed, disseminated	Fever, abdominal pain, weakness; hepatomegaly; severe metabolic acidosis with extensive skeletal involvement	21	Normal	Yes	Bicarbonate, supportive care	MCP842 protocol chemotherapy for NHL	Lactic acidosis resolved after chemotherapy; recovered despite tumor lysis complications and catheter-related Acinetobacter infection
Luscri et al. (2009) [25]	7/M	B-cell precursor ALL	Second relapse after hematopoietic cell transplant	Fever, leukemic relapse, refractory hypoglycemia, lactic acidosis, respiratory failure, and acute renal failure	>30	25-33	Yes	Continuous dextrose infusion, bicarbonate infusion, vitamin B supplementation (thiamine/riboflavin), hemodialysis	No further chemotherapy due to poor prognosis; supportive care only	Progressive deterioration; care withdrawn; died on day 9
Rastogi et al. (2008) [26]	11/M	Burkitt lymphoma, HIV	Newly diagnosed	HIV/AIDS, cerebral palsy, and seizure disorder. Developed persistent severe hypoglycemia and lactic acidosis 12 days after diagnosis of Burkitt lymphoma. Hypoglycemia refractory to glucose infusion	15.6	20-40	Yes	Bicarbonate, continuous glucose infusion, glucagon	Chemotherapy for Burkitt lymphoma	Despite metabolic support, patient died 1 month after admission due to complications of underlying illness
Hayek and Srinivasan (2003) [27]	7/M	Pre-B-cell ALL	Newly diagnosed	Polydipsia, polyuria, approximately 10-kg weight loss; pallor, cervical lymphadenopathy, mild hepatosplenomegaly; anemia and renal tubular dysfunction	8.4	96	Yes	Alkaline therapy; 48 h of peritoneal dialysis	Chemotherapy for pre-B-cell ALL	Lactic acidosis resolved within 2 days of chemotherapy; renal size and function normalized; complete response
Révész et al. (1995) [28]	8/M	Burkitt lymphoma	Relapsed disease with renal involvement	Severe metabolic acidosis, renal enlargement, renal diabetes insipidus; lymphomatous kidney infiltration	24	Normal	Yes	Sodium bicarbonate, IV thiamine	Chemotherapy + renal radiotherapy	Temporary lactate improvement followed by progressive disease; died 4 months after relapse
Ali et al. (1994) [29]	12/M	B-cell precursor ALL	Newly diagnosed, with later multiple relapses	Polyuria, polydipsia, weight loss, abdominal pain; severe anion gap metabolic acidosis with bilateral renal enlargement	12	Normal	Yes	Alkaline therapy and electrolyte correction	Intensive ALL chemotherapy; later salvage therapy and HSCT	Lactic acidosis and renal enlargement resolved with chemotherapy; died after second HSCT from infectious complications
Coleman et al. (1982) [30]	3/M	Sacrococcygeal teratocarcinoma with hepatic metastases	Progressive disease during chemotherapy	Cachexia, massive hepatomegaly, ascites, tachypnea, ketonuria, severe metabolic acidosis	15.8	Normal	Yes	Sodium bicarbonate, thiamine, insulin + glucose infusion	Vinblastine and bleomycin	Temporary improvement with insulin therapy; discharged; died 6 weeks later from progressive malignancy
Coleman et al. (1982) [30]	2/M	T-cell ALL	Fourth marrow relapse with hyperleukocytosis	Tachypnea, lymphadenopathy, splenomegaly, extreme leukocytosis, severe lactic acidosis, ketolactic state	13.8	Normal	Yes	Thiamine, insulin infusion with glucose; bicarbonate avoided	Methylglyoxal-bis-guanylhydrazone attempted for remission	Transient lactate improvement; rapidly deteriorated with neurologic event; died
Field et al. (1966) [31]	4/M	ALL	Relapse	Dyspnea, lethargy, Kussmaul respiration, severe metabolic acidosis	20.2	NR	Yes	Bicarbonate, supportive care	Hydroxyurea, mediastinal radiotherapy, hydrocortisone, dexamethasone	Improved. Lactic acidosis resolved with rapid cytoreduction; died ~1 month later from progressive leukemia
Field et al. (1966) [31]	8/M	ALL	Newly diagnosed	Metabolic acidosis, elevated CSF pressure, progressive illness	12.5	NR	Yes	Bicarbonate, supportive care	Methotrexate discontinued; vincristine initiated	Improved; later died from overwhelming pneumonia

The management of malignancy-associated type B lactic acidosis remains challenging as currently available supportive therapies do not address the underlying mechanism of excessive lactate production. Intravenous glucose, bicarbonate replacement, renal replacement therapy, and ventilatory support may transiently stabilize metabolic abnormalities but generally fail to reverse ongoing tumor glycolysis. Thiamine supplementation has been administered empirically to facilitate pyruvate dehydrogenase function and shift pyruvate metabolism toward oxidative phosphorylation, although evidence supporting its clinical benefit remains limited [16]. Likewise, dichloroacetate (DCA) has been proposed as a metabolic modulator through its ability to inhibit pyruvate dehydrogenase kinase, thereby shifting to aerobic metabolism and reducing lactate production; however, published experience remains restricted to isolated case reports and small series, precluding routine clinical use [17].

Despite timely corticosteroid cytoreduction, subsequent salvage therapy with inotuzumab ozogamicin, and maximal intensive care support, our patient demonstrated persistent hyperlactatemia that closely paralleled refractory leukemia, ultimately progressing to multiorgan failure and death. This clinical course is consistent with published experience, in which failure to achieve disease control is frequently accompanied by persistent metabolic derangement and poor survival, underscoring that hyperlactatemia in this setting reflects ongoing tumor metabolism rather than failure of supportive care alone [18].

Serum lactate levels closely mirrored disease burden throughout the clinical course in this patient, rising in concert with leukemic blast proliferation and falling transiently following corticosteroid-mediated cytoreduction. This observation supports the proposition that serial lactate measurement may serve as a surrogate biomarker for tumor metabolic activity in children with leukemia and suspected Warburg physiology, complementing conventional markers of disease response such as peripheral blast count and bone marrow assessment. Notably, the concurrent fever, cytopenias, and hyperferritinemia in this patient prompted consideration of hemophagocytic lymphohistiocytosis (HLH); however, the absence of response to immunosuppressive therapy and the predominance of a metabolic derangement profile, in the absence of classical HLH trigger features, supported the Warburg-driven mechanism as the primary etiology rather than a secondary HLH process.

The administration of inotuzumab ozogamicin in this patient warrants specific comment given its known hepatotoxic potential. Inotuzumab ozogamicin is associated with a clinically significant risk of sinusoidal obstruction syndrome or veno-occlusive disease (SOS/VOD), a hepatic complication that can impair lactate clearance and contribute to secondary lactic acidosis. In the present case, however, hepatic function parameters remained relatively preserved throughout the peri-inotuzumab period, and metabolic derangements, including persistent hyperlactatemia and recurrent hypoglycemia, clearly preceded inotuzumab initiation, making inotuzumab-associated SOS/VOD an unlikely contributor to the observed metabolic syndrome. Clinicians prescribing inotuzumab ozogamicin in patients with pre-existing metabolic acidosis or concurrent leukemia-associated lactic acidosis should nonetheless remain vigilant for this potentially compounding complication and monitor hepatic function indices and lactate levels longitudinally [19].

## Conclusions

Malignancy-associated type B lactic acidosis should be considered in children with relapsed or refractory leukemia who develop persistent hyperlactatemia with refractory hypoglycemia after more common etiologies have been excluded. This case illustrates that these unexplained metabolic abnormalities should raise suspicion for evolving refractory disease and prompt timely reassessment of disease status. Although supportive measures remain important, effective cytoreduction continues to represent the only intervention consistently associated with reversal of this rare but frequently fatal metabolic complication.
